# Direct observation of glycans bonded to proteins and lipids at single molecule level

**DOI:** 10.1126/science.adh3856

**Published:** 2023-10-12

**Authors:** Kelvin Anggara, Laura Sršan, Thapakorn Jaroentomeechai, Xu Wu, Stephan Rauschenbach, Yoshiki Narimatsu, Henrik Clausen, Thomas Ziegler, Rebecca L. Miller, Klaus Kern

**Affiliations:** 1 Max-Planck Institute for Solid-State Research; Stuttgart, DE-70569, Germany; 2 Institute of Organic Chemistry, University of Tübingen; Tübingen, DE-72076, Germany; 3 Copenhagen Center for Glycomics, Department of Cellular & Molecular Medicine, University of Copenhagen; Copenhagen, DK-2200, Denmark; 4 Chemistry Research Laboratory, Department of Chemistry, University of Oxford; Oxford, OX1 3TA, United Kingdom; 5 GlycoDisplay ApS, Copenhagen, DK-2200, Denmark; 6 Institut de Physique, École Polytechnique Fédérale de Lausanne; Lausanne, CH-1015, Switzerland

## Abstract

Proteins and lipids decorated with glycans are found throughout biological entities, playing roles in biological functions and dysfunctions. Current analytical strategies for these glycan-decorated biomolecules, termed glycoconjugates, rely on ensemble averaged methods that do not provide a full view of positions and structures of glycans attached at individual sites in a given molecule, especially for glycoproteins. Here we show single molecule analysis of glycoconjugates by direct imaging of individual glycoconjugate molecules using low-temperature scanning tunneling microscopy. Intact glycoconjugate ions from electrospray are soft-landed on surface for their direct single molecule imaging. The sub-molecular imaging resolution corroborated by quantum mechanical modeling unveils the entire structures and attachment sites of glycans in glycopeptides, glycolipids, N-glycoproteins, and O-glycoproteins densely decorated with glycans.

Glycan (also known as carbohydrate) is one of the four essential organic building blocks found in all forms of life (1–7). Glycans play key roles in cellular functions (7, 8), growth and development (2, 9), identification (2–4), shapes (10, 11), and energy storage (12). In biological systems, glycans are predominantly found attached to other biomolecules such as proteins and lipids. These glycan-decorated biomolecules, termed glycoconjugates, are produced via complex enzymatic glycosylation events – the most common and diverse post translational modification (PTM) that greatly expands the functions of biomolecules (13, 14). The abundance of glycoconjugates in biological systems and their roles in health and disease make them attractive targets in basic and translational research for new therapeutic and diagnostic strategies (1–3, 15, 16). However, despite the ubiquity and importance of glycoconjugates, research to unveil their structure-property relationships has been challenging (17–19).

Glycoconjugates possess extensive structural heterogeneity (i.e. multiple variants of sequence) and structural isomerism (i.e. structures with equal masses), which pose a challenge for today’s analytical methods (17–19). Glycoconjugates are presently analyzed by a combination of chemical labeling, chemoenzymatic digestion, and ensemble averaged methods to indirectly obtain the most likely structures present in a sample (18, 19). Ensemble averaged analysis of structurally heterogeneous and isomeric glycoconjugates however obscures the position and structures of glycans bonded to a biomolecule, particularly for proteins with multiple glycans attached. As a result, insights into the structures of individual molecules are lost with ensemble averaged analysis, which hinders structure-property relationship studies of glycoconjugates. Preventing the loss of structural information for individual molecules requires glycoconjugate molecules to be analyzed at single molecule level.

Here we realize single molecule analysis of glycoconjugates by performing direct, label-free, spatial imaging on individual glycoconjugate molecules. We show that imaging single glycoconjugates at sub-nanometer resolution reveals the primary structure of each molecule, by unveiling how its constituent amino acid, lipid, and monosaccharide subunits connect to one another. As a result, our imaging method establishes glycan sequences at every glycan attachment site in a glycoconjugate by locating every monosaccharide in the glycoconjugate molecule discriminated by its stereoconfiguration and side group. We demonstrate our method for a wide range of glycoconjugates, starting from simple glycopeptides and glycolipids, up to complex glycoproteins with more than 20 attached glycans. Our work shows that single molecule imaging provides a direct access to all glycan structures bonded to the complex glycopeptides, glycolipids, and glycoproteins at single molecule level.

## Direct imaging of glycoconjugates soft-landed at a surface

**Fig. 1:**
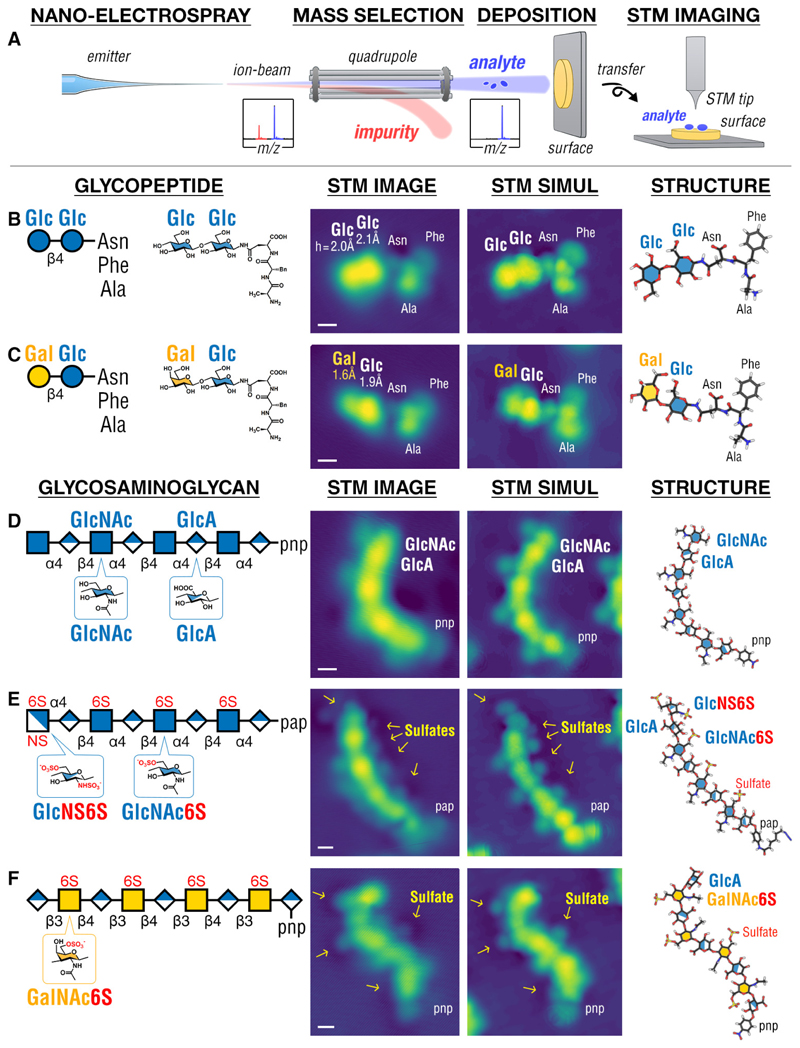
STM imaging of simple glycoconjugates and glycosaminoglycans (GAGs). (**A**) Schematic of the experiment: glycoconjugate or GAG ions generated by nESI were mass-selected, soft-landed intact on surface held at 120 K, and imaged by STM at 11 K (see Methods). STM images of glycopeptides, Glc-Glc-AsnPheAla (**B**) and Gal-Glc-AsnPheAla (**C**), reveal the glycan and the peptide domains of the molecule and differentiate each monosaccharide in the glycan domain i.e. glucose (Glc) vs galactose (Gal). Imaging GAGs (**D-F**) reveals the positions of N-Acetyl groups on all N-acetylglucosamine (GlcNAc) monosaccharides, differentiating them from the glucuronic acid (GlcA) monosaccharides as well as the sulfated GlcNAc6S, GalNAc6S, and GlcNS6S monosaccharides. The GAGs in (**D**) and (**F**) are terminated by para-nitrophenyl (pnp), while in (**E**) the GAG is terminated by para-(6-azidohexanamido)phenyl (pap). STM images were interpreted by STM simulation of molecular structures computed by DFT. Scale bar is 0.5 nm. Glycan icons follow the SNFG standard (56).

We accomplished direct imaging of single glycoconjugate molecules by combining soft-landing electrospray ion beam deposition (ESIBD) (20, 21) and scanning tunneling microscopy (STM) (see Methods for details). We show STM imaging of single glycoconjugates on Cu-surface at cryogenic temperatures corroborated by Density Functional Theory (DFT) calculations provides direct access to their structural information (Fig. 1). We imaged glycoconjugate ions obtained from nanoelectrospray ionization (nESI) (22) (Fig. S1), whose usage was critical to lower the amount of sample required to ~1 nanomole, given that glycoconjugates, unlike proteins (23, 24) and glycans (25), are more limited in sample quantity. In cases of sulfated molecules (Fig. S2), we deposited the molecules on a more inert Ag-surface to preserve the labile sulfate groups on surface (Fig. S3).

We first highlight the capabilities of STM imaging and DFT modeling in characterizing monosaccharide structures of glycans. We imaged two glycopeptides (26) (Fig. 1b,c) composed of a disaccharide (cellobiose, Glcβ1–4Glc, or lactose, Galβ1–4Glc) linked to a tripeptide, and three glycosaminoglycans (GAGs) (Fig. 1d-f). STM imaging of the glycopeptides was found to differentiate the glycan and peptide moieties by their heights: tall/bright for the glycan and low/dim for the peptide (Fig. 1b,c), which allowed the DFT calculations to yield the primary structure of the glycopeptides, resolving the order by which amino acid and monosaccharide subunits in the molecule are connected to one another. In addition, STM imaging was found to discriminate glucose from its epimer, galactose (i.e. they differ only in the stereoconfiguration of their C4-atoms), given that the glucose (*h* = 2.1 ± 0.2 Å, *N* = 45) was observed consistently taller than the galactose (*h* = 1.7 ± 0.3 Å, *N* = 75). STM imaging was also found to locate and identify side groups and sulfate moieties present in every monosaccharide, as exemplified by the imaging of GAGs (Fig. 1d-f). Each GlcNAc monosaccharide was observed with a dim protrusion corresponding to the N-Acetyl (NAc) moiety, clearly distinct from the GlcA monosaccharides (Fig. 1d). Interestingly, each GlcNAc6S and GalNAc6S monosaccharide was observed with its sulfate moiety (a dim protrusion encircled with a dark ring) on the opposite side from its NAc moiety (a dim protrusion without dark ring) (Fig. 1e,f); whereby GlcNS6S was observed with two sulfate moieties, each appearing as a dim protrusion with a dark ring (Fig. 1e). We further ascertained the STM appearance of sulfate moiety on the same surface as a dim protrusion encircled with a dark ring by imaging simple aryl sulfates (Fig. S3). Our findings show that STM imaging and DFT modeling have sufficient sensitivity and resolution to locate glycans in molecules, and discriminate the constituent monosaccharides based on their stereoconfigurations and side groups.

**Fig. 2:**
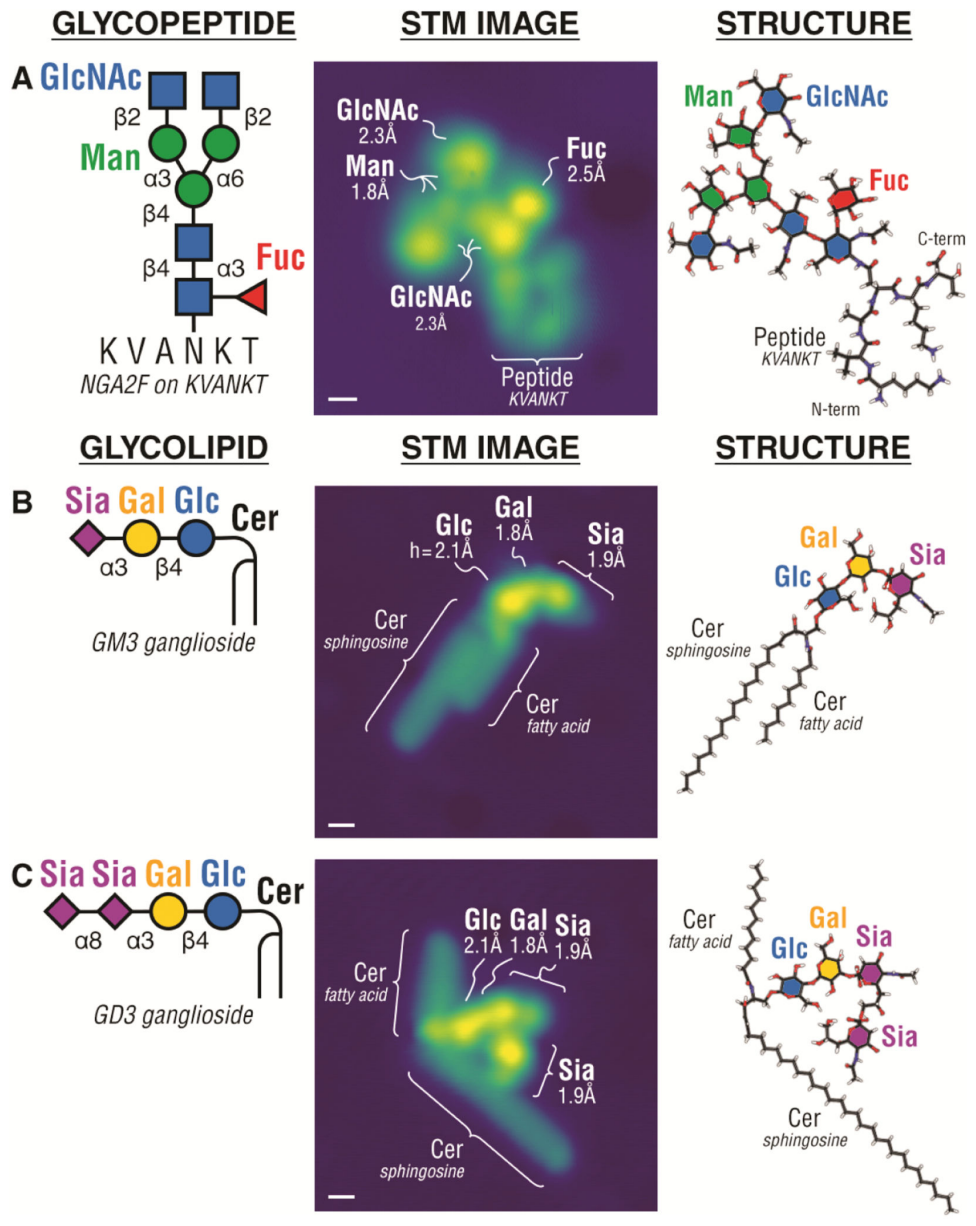
STM imaging of single glycopeptides and glycolipids. STM images of an N-glycopeptide (biantennary N-glycan NGA2F on the KVANKT peptide) (**A**), and glycolipids, GM3 ganglioside (**B**) and GD3 ganglioside (**C**), reveal the glycan, peptide, and ceramide (Cer) domains in the respective molecules (Cer consisted of varying length fatty acid chains and sphingosine). STM imaging differentiates individual monosaccharides in the glycoconjugate i.e. glucose (Glc), galactose (Gal), sialic acid (Sia = Neu5Ac), N-acetylglucosamine (GlcNAc), mannose (Man), and fucose (Fuc). STM images were interpreted by structures computed by DFT. Scale bar is 0.5 nm.

We show the perspectives of our direct single molecule analysis by determining structures of entire glycans present in complex glycopeptides, glycolipids, and glycoproteins at single molecule level. For glycopeptides, we examined an egg yolk sialoglycopeptide derivative (Fig. 2a), which is widely used in biochemical applications (27). For glycolipids, the GM3 and GD3 gangliosides (Fig. 2b,c) were chosen due to their roles as cancer antigens (5, 15). For N-glycoproteins, we chose the widely studied pancreatic RNase B (28). Finally, as a representative for O-glycoproteins, we chose a fragment of human mucin MUC1, one of the most complex glycoproteins in biological systems, which is also overexpressed with aberrant O-glycans in cancer, and a promising cancer biomarker and immunotherapeutic target (29, 30). In all cases, there is a clear height contrast between the bright glycan domain and the dim peptide or lipid domains that establishes their respective primary structures. For the N-glycopeptide (Fig. 2a), the height contrast discriminated the GlcNAc and Fuc monosaccharides (*h* = 2.2 ± 0.4 Å, *N* = 132) from the mannose (*h* = 1.9 ± 0.4 Å, *N* = 66); while, in the glycolipids (Fig. 2b,c), the height contrast differentiated glucose (one lobe, *h* = 2.1 ± 0.3 Å, *N* = 158), galactose (one lobe, *h* = 1.9 ± 0.2 Å, *N* = 158), and sialic acid (Neu5Ac) (two or more lobes) from one another. The imaging was found to distinguish glycosidic bonds by the characteristic angle formed between monosaccharides when their pyranose rings adsorb horizontally on surface (experimentally verifiable by their respective heights). For example, the Siaα2–3Gal β1–4Glc in GM3 and GD3 (Sia = Neu5Ac monosaccharide) was observed to form an obtuse 141 ± 22º angle (*N* = 158), while the Glcβ1–4Glcβ1–4Glc in cellohexaose was observed to form a straight 180 ± 25º angle (*N* = 204) (31). In addition, the imaging of single glycolipids revealed their molecular conformations (Fig. S4) and allowed discrimination of the ceramide moiety with varied lipid chain lengths (Fig. S5), both of which may provide additional information towards structural studies of lipids. Interestingly, we observed the ‘open’ conformation of the lipid moiety in glycolipids (Fig. 2c and S4), which has been discussed in relation to mechanisms of membrane fusions and protein-membrane interactions (32, 33).

## Imaging single N- and O-glycoproteins

**Fig. 3:**
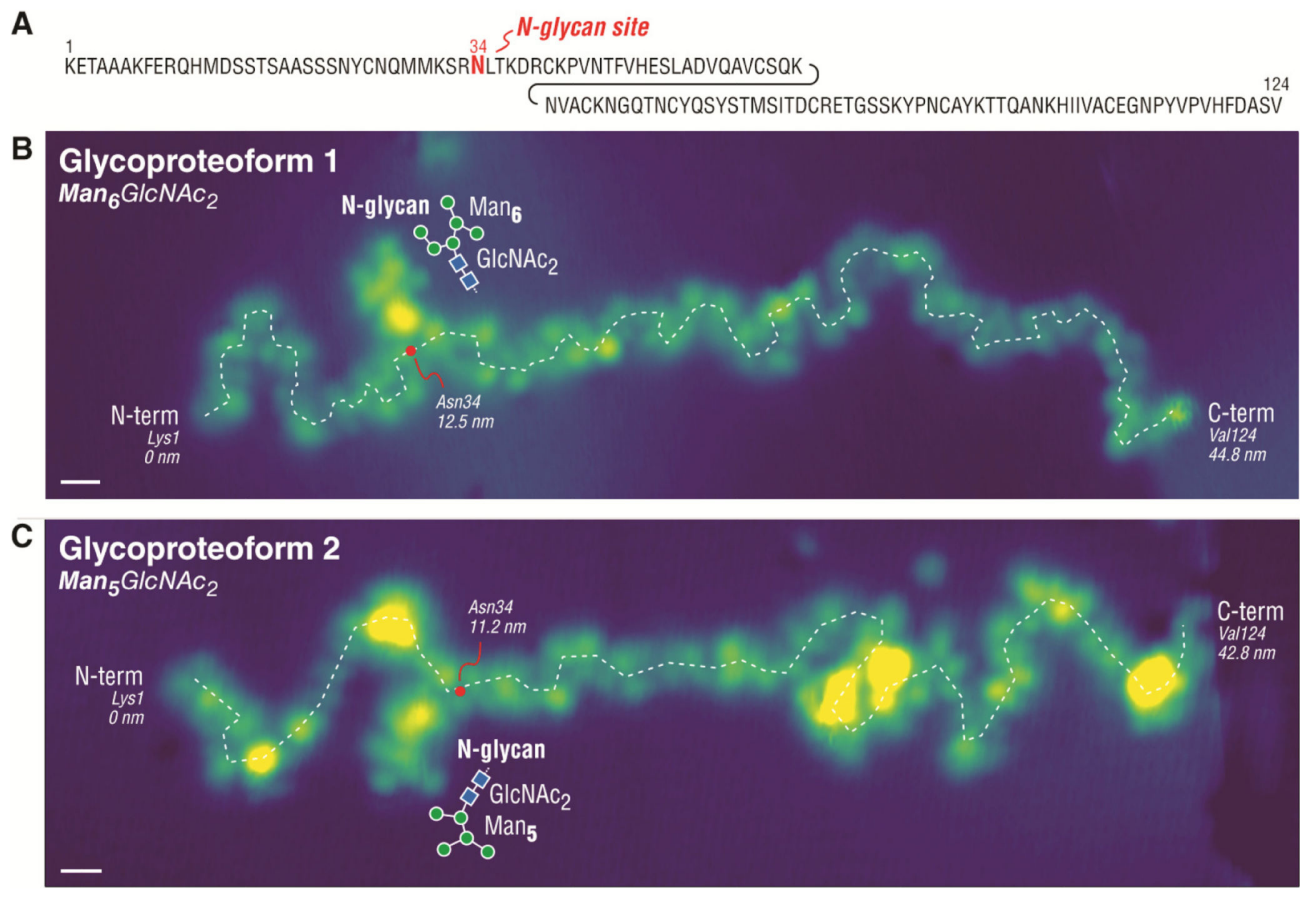
STM imaging of single N-glycoproteins. (**A**) Sequence of RNase B (124aa) with one N-linked glycan at Asn34. Imaging single unfolded RNase B molecules reveals the N-glycan position along the protein backbone and the N-glycan structure found on individual glycoproteoforms one-molecule-at-a-time, as shown in (**B**) for Man_6_GlcNAc_2_ and in (**C**) for Man_5_GlcNAc_2_. The position of Asn34 is estimated by a red dot along the protein backbone (white dashed line). Scale bar is 1 nm.

Direct imaging of single a N-glycoprotein, RNase B, revealed the structure and the location of the N-glycan bonded to the protein backbone (28) (Fig. 3). We examined RNase B by imaging individual proteins in their fully unfolded state, which we prepared by exclusively depositing the highly charged protein ions on surface (24) (Fig. S6). Given that RNase B has five glycoproteoforms (28) (each featuring one of five distinct N-glycan structures from Man_5_GlcNAc_2_ to Man_9_GlcNAc_2_), our single protein imaging allowed the glycoproteoforms of RNase B to be determined one-molecule-at-a-time, as shown in Figure 3b for Man_6_GlcNAc_2_ and in Figure 3c for Man_5_GlcNAc_2_ – the two most abundant glycoproteoforms of RNase B (Fig. S1g). The STM imaging clearly revealed the glycan attachment site by locating the intersection between the N-glycan and the protein backbone (red dots in Fig. 3b,c). Analysis of 33 individual N-glycoproteins confirmed residue 34 (± 2) as the glycan attachment site, consistent with Asn34 as the known glycan attachment site for RNase B (28).

**Fig. 4:**
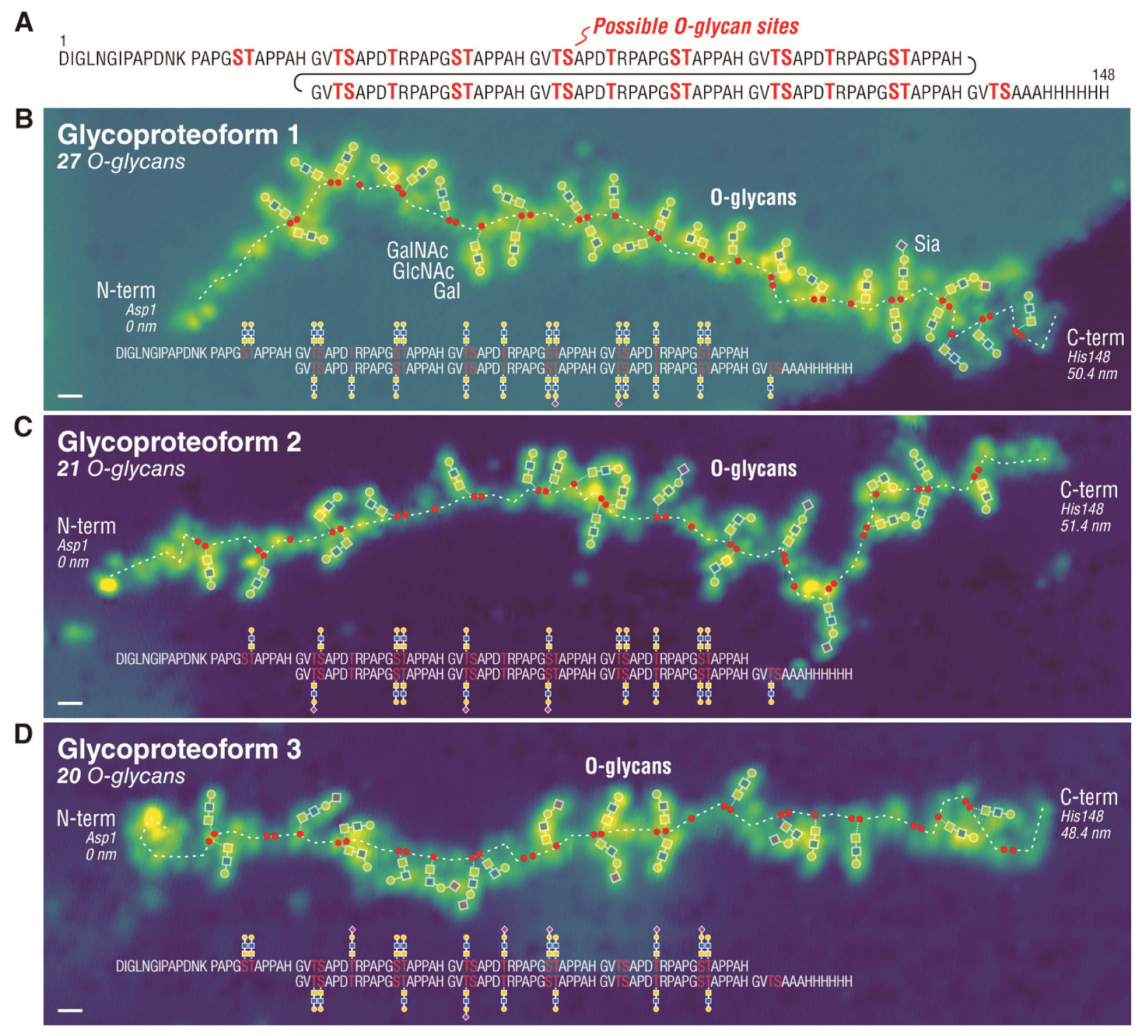
STM imaging of single O-glycoproteins. (**A**) Sequence of MUC1 reporter (148aa) containing 6.5 tandem repeats of 20 amino acids (GV**TS**APD**T**RPAPG**ST**APPAH) decorated with O-glycans at the Ser (**S**) and Thr (**T**) residues (total of 34 potential O-glycosites). Imaging single MUC1 proteins reveals the number, the structure, and the attachment site of O-glycans decorating the protein, as shown for a glycoproteoform with 27 O-glycans in (**B**), 21 O-glycans in (**C**), and 20 O-glycans in (**D**). The positions of S and T residues are indicated by red dots along the protein backbone (white dashed line). The unannotated STM images are given in Fig. S8. Scale bar is 1 nm.

To illustrate the full perspective of single glycoprotein imaging, we examined an O-glycoprotein fragment derived from the large mucin MUC1. Mucins are considered one of the last frontiers in glycoanalytics that has remained unexplored to a large extent (34–36), despite their wide importance in mucosal biology and host-pathogen interactions (37). Structural analysis of mucins and their multiple glycosylation sites is challenging due to their enormous size and dense decoration of O-glycans, resulting in heterogeneity and resistance to protease digestion (34–36). Here we show that it is possible to analyze such heavily O-glycosylated proteins one-molecule-at-a-time (Fig. 4) by using the soft deposition and imaging of single glycoproteins on surface (Fig. S7).

We imaged a representative fragment of the densely O-glycosylated tandem repeat region of human MUC1 mucin as an O-glycoprotein reporter with relatively homogeneous trisaccharide O-glycans (ie. ‘core 3’: Galβ1-4GlcNAcβ1-3GalNAcα1-O-S/T) (Fig. 4). For this, we employed our recently developed cell-based strategy using genetically glycoengineered HEK293 cells for recombinant production of mucin reporter glycoproteins with custom-designed O-glycosylation (35) (Fig. 4a) (see Methods). The MUC1 O-glycoprotein reporter, featuring 34 potential O-glycosylation sites, was analyzed by intact and bottom-up mass spectrometry, and profiling of released O-glycans, which revealed a relatively homogeneous mixture of O-glycans (mainly ‘core 3’) and number of O-glycans (mainly 19 – 23 glycans) (35, 38) (see also Fig. S1h and Fig. S9 for MUC1 sample used in single molecule imaging experiments).

Imaging single MUC1 O-glycoproteins allows direct observation of the variation in number, structure, and attachment sites of O-glycans on the protein backbone, as shown in examples with 27, 21, and 20 O-glycans (Fig. 4b,c,d). On the individual MUC1 molecules, we found the O-glycans mainly to be the ‘core 3’ trisaccharides with the occasional sialylated ‘core 3’ tetrasaccharide (Siaα2-3Galβ1-4GlcNAcβ1-3GalNAc) in agreement with the glycoprofiling analysis (35, 38) (Fig. S9). Most importantly, the direct imaging of MUC1 clearly revealed the positions of each O-glycan at S and T sites along the protein (red dots in Fig. 4b,c,d) (see Fig. S10 for an example). Analysis of the O-glycan positions on 18 MUC1 O-glycoproteins observed (Table S1) revealed three prevalent patterns of O-glycan distribution on the MUC1 tandem repeats as -**TS**-T-**ST**- ; -**T**S-**T**-**ST**- ; and -**T**S-T-**ST**- (bold underlined indicates glycosylated, see Table S2). These patterns are largely in agreement with the predicted O-glycosylation sequence of the MUC1 from both in vitro (39) and in vivo (38) enzyme specificity analysis. The O-glycosylation process is a complex event with multiple isoenzymes (polypeptide GalNAc-transferases) each attaching O-glycans at select positions in proteins, and the O-glycosylation of the five possible sites in the MUC1 tandem repeat requires sequential orchestrated action of multiple isoenzymes (39). Further analysis of the STM results (Table S1) revealed on average 3.4 O-glycans per tandem repeat with preferred positions at T in VTSA (87% occupied) and ST in GSTA (78% and 83% occupied respectively) and less preferred positions at S in VTSA (44% occupied) and T in PDTR (55% occupied). These results corroborate our previous studies of the MUC1 reporter protein with ‘core 3’ O-glycans (35, 38), for which we found reduced occupancy of O-glycans at S in VTSA and T in PDTR (38, 40, 41). Direct STM imaging thereby yields detailed snapshots of single molecule glycoproteoforms that can unveil potential interplay between glycosylation at different positions in proteins and the glycan structures that may be assembled at these positions (Table S2). In addition, STM imaging allows direct observation of glycan-glycan interactions dictating the overall shape of the protein backbone. We expect the single molecule analysis approach to be widely applicable to glycoproteins that can be electrosprayed in unfolded states, regardless of size and numbers of attached glycans. In case of increasingly dense glycans causing proteins to unfold incompletely, the STM tip could be used to further unfold the protein to clarify its primary structure (Fig. S11).

## Conclusion

Our combination of electrospray deposition and scanning tunnelling microscopy analysis provides an opportunity to look directly at the primary structures of complex glycoconjugates, including glycoproteins with multiple glycans attached. This technology, corroborated by DFT modeling, should enable direct observation of diverse post-translational modifications (PTMs) on biomolecules (42, 43), as well as structures of glycoconjugates that are well beyond today’s analytical capabilities, such as proteoglycans (44), glycoRNAs (45), lipopolysaccharides (46), and carbohydrate vaccines (16). While the present work demonstrates that prior knowledge of the amino acid sequence of the glycoproteins is advantageous to enable interpretation of the STM images, we recognize that STM imaging can still be further improved to identify each amino acid and monosaccharide in a molecule and thus identify single proteins/glycoproteins in complex biological mixtures. These improvements include the use of a functionalized tip to resolve and distinguish covalent bonds in a molecule (47), as well as the use of tunneling spectroscopy (48), nuclear spin detection (49), or optical fingerprinting (50–52) to identify electronic signatures of specific atoms or functional groups in molecules. With these improvements, we expect STM can contribute to identification of unknown glycoproteins or glycolipids, which may ultimately lead to the discovery of individual glycoproteins and glycolipids in a complex cellular mixtures, particularly in the context of glycoproteomics and glycolipidomics studies. Complementing these improvements with automated structure solvers (53–55) or tip preparation will increase the throughput of scanning probe microscopy and create opportunities to solve previously intractable problems in single molecule (bio)analytical chemistry.

## Supplementary Material

Supplementary Material

## Data Availability

All data are available in the main text or the supplementary materials. Raw STM images and DFT computed structures are available at the Data Repository of the Max Planck Society (57).

## One sentence summary

Single molecule imaging of glycan-decorated proteins and lipids unveils all glycan structures bonded to the host molecules
