# Supplementary material for: Direct observation of glycans bonded to proteins and lipids at single molecule level
Source: Science. Author manuscript; Available in PMC 2023 Oct 20. (PMC7615228; doi:10.1126/science.adh3856)
Supplement: Supplementary Material [file EMS189541-supplement-Supplementary_Material.docx]

**Supplementary Figures**


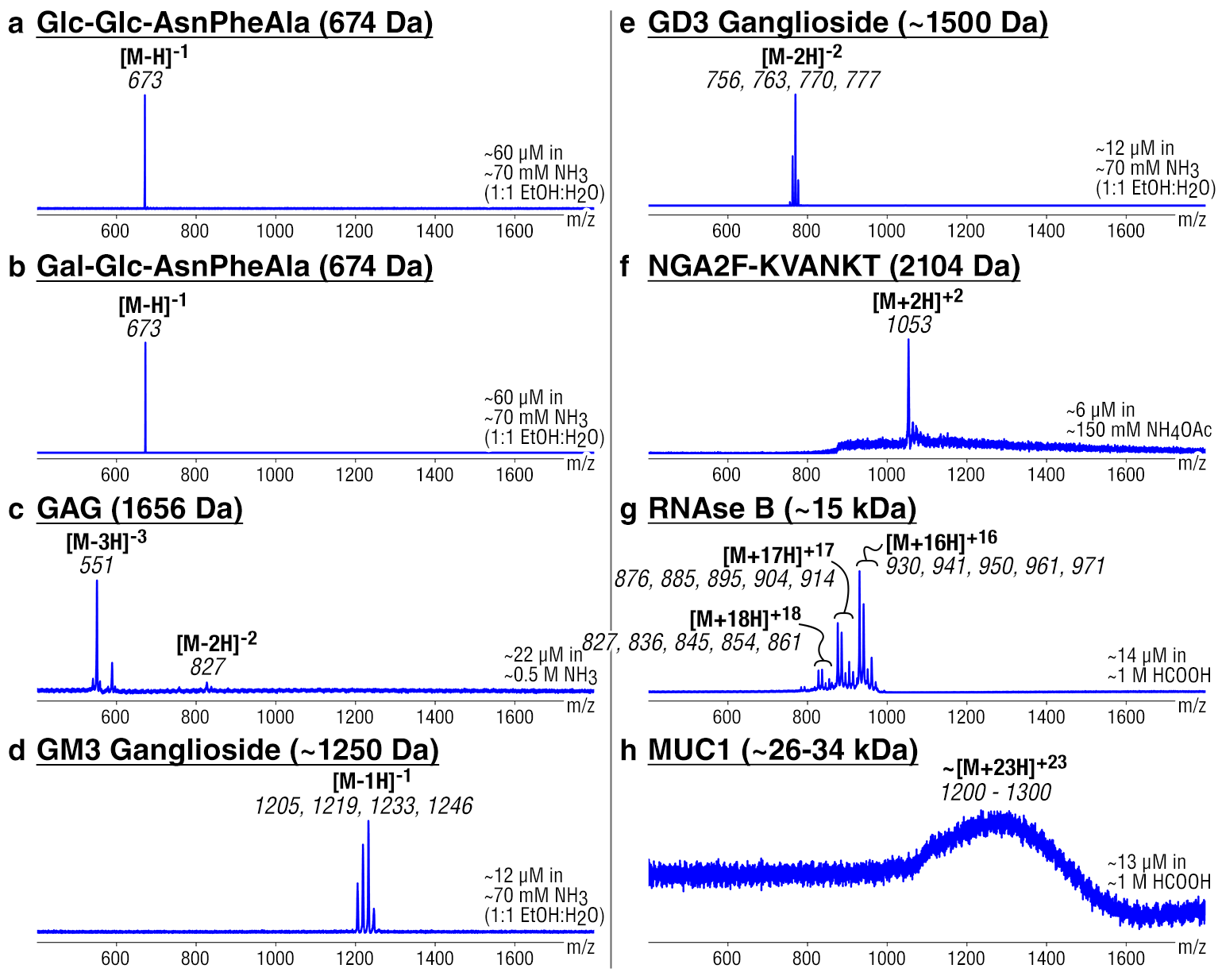


**Fig. S1 | Time-of-flight mass spectra of glycoconjugate ions deposited on Cu(100) surface by ESIBD.** The mass spectra show the composition of the mass-selected ion beam used for the soft molecular deposition on the Cu-surface. The mass spectra are given for Glc-Glc-AsnPheAla in (**a**), Gal-Glc-AsnPheAla in (**b**), GAG (GlcNAc-GlcA-GlcNAc-GlcA-GlcNAc-GlcA-GlcNAc-GlcA-pnp, pnp = para-nitrophenyl) in (**c**), GM3 ganglioside in (**d**), GD3 ganglioside in (**e**), NGA2F-KVANKT glycopeptide in (**f**), RNase B in (**g**), and MUC1 in (**h**). Multiple peaks separated by m/z 14 for GM3 (**d**) and m/z 7 for GD3 (**e**) were observed due to multiple lengths (i.e. total of CH_2_ units) of lipid chain present in the GM3 and GD3 samples. Repeated sets of five peaks were observed for RNase B (**g**) due to the five glycoproteoforms of the protein present in the sample, ranging from Man_5_GlcNAc_2_ to Man_9_GlcNAc_2_. No prominent peaks were observed for MUC1 (**h**) due to the high structural heterogeneity of the glycoprotein. Solvent used in the spray solutions was water, unless indicated otherwise.


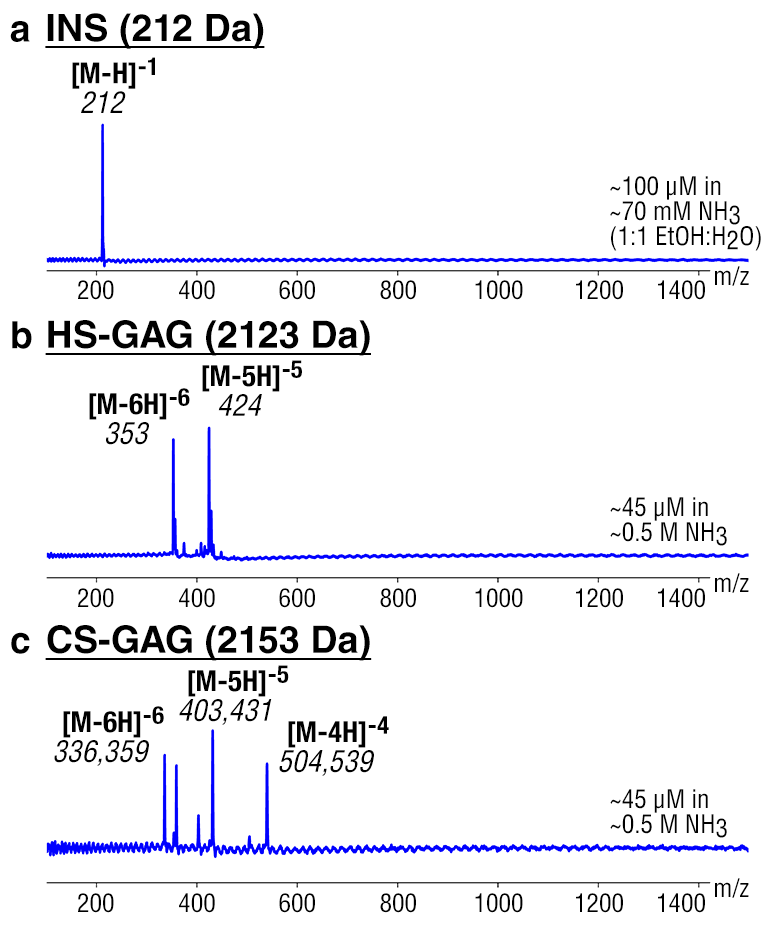


**Fig. S2 | Time-of-flight mass spectra of sulfated ions deposited on Ag(110) surface by ESIBD.** Similar to Fig. S1, the mass spectra characterizes the mass-selected ion beam used for the soft molecular deposition on the Ag-surface. The mass spectra are given for indoxyl sulfate (INS) in (**a**), HS-GAG in (**b**), and CS-GAG in (**c**). HS-GAG is GlcNS6S-GlcA-GlcNAc6S-GlcA-GlcNAc6S-GlcA-GlcNAc6S-GlcA-pap and CS-GAG is GlcA-GalNAc6S-GlcA-GalNAc6S-GlcA-GalNAc6S-GlcA-GalNAc6S-GlcA-pnp, where pap = para-(6-azidohexanamido)phenyl, and pnp = para-nitrophenyl. The lighter satellite peaks observed for CS-GAG (m/z = 336, 403, and 504) correspond to the CS-GAG ions that lost their pnp tag. Solvent used in the spray solutions was water, unless indicated otherwise.


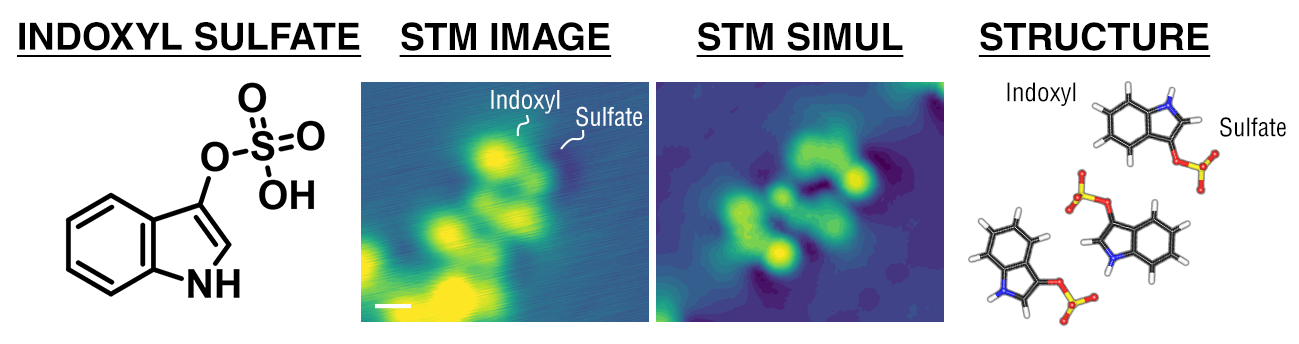


**Fig. S3 | STM appearance of sulfates on Ag(110) surface.** The intact sulfate in indoxyl sulfate (INS) was imaged as a dim protrusion (~0.6 Å tall) encircled by a dark depression (~0.1 Å deep), as confirmed the STM simulations from *ab initio* calculations. INS were observed to form a two-dimensional assembly on the surface when they were soft landed on ~120 K Ag(110) surface with ~0.4 eV landing energy. Scale bar is 0.5 nm.


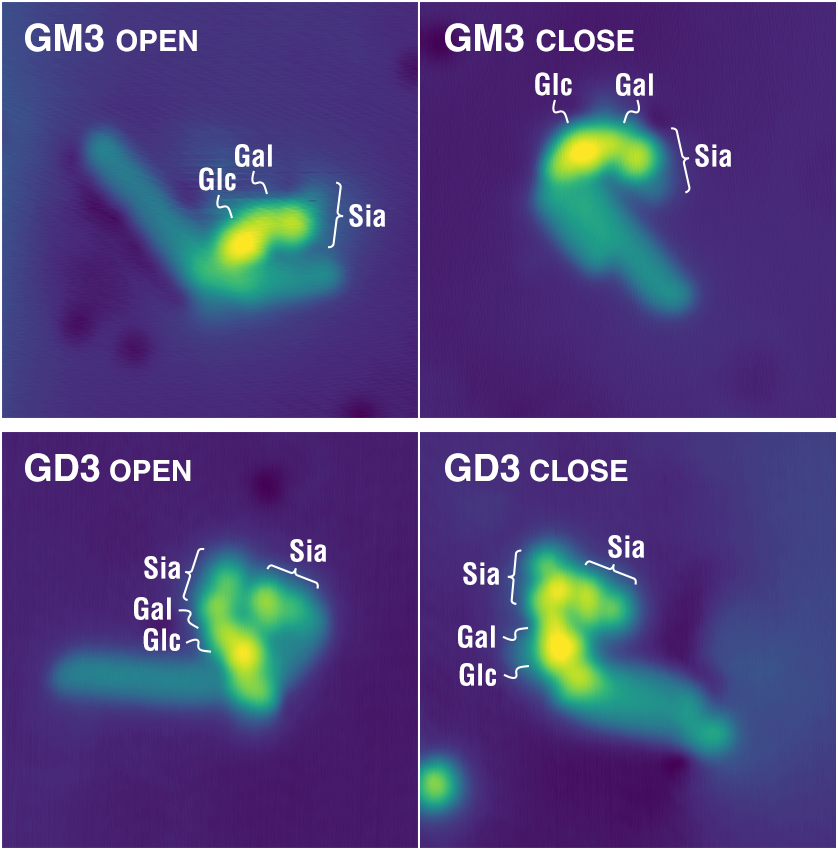


**Fig. S4 | Conformations of glycolipids on surface.** GM3 and GD3 glycolipids deposited on surface were observed with their respective lipid chains in either ‘close’ (i.e. two polyalkane chains appear next to one another) or ‘open’ (i.e. two polyalkane chains appear separate from one another) conformations. For GM3, we observed 34 cases of ‘open’ and 57 cases of ‘close’, wherein for GD3, we observed 10 cases of ‘open’ and 57 of ‘close’. Each STM image is 8 × 8 nm^2^ in size.


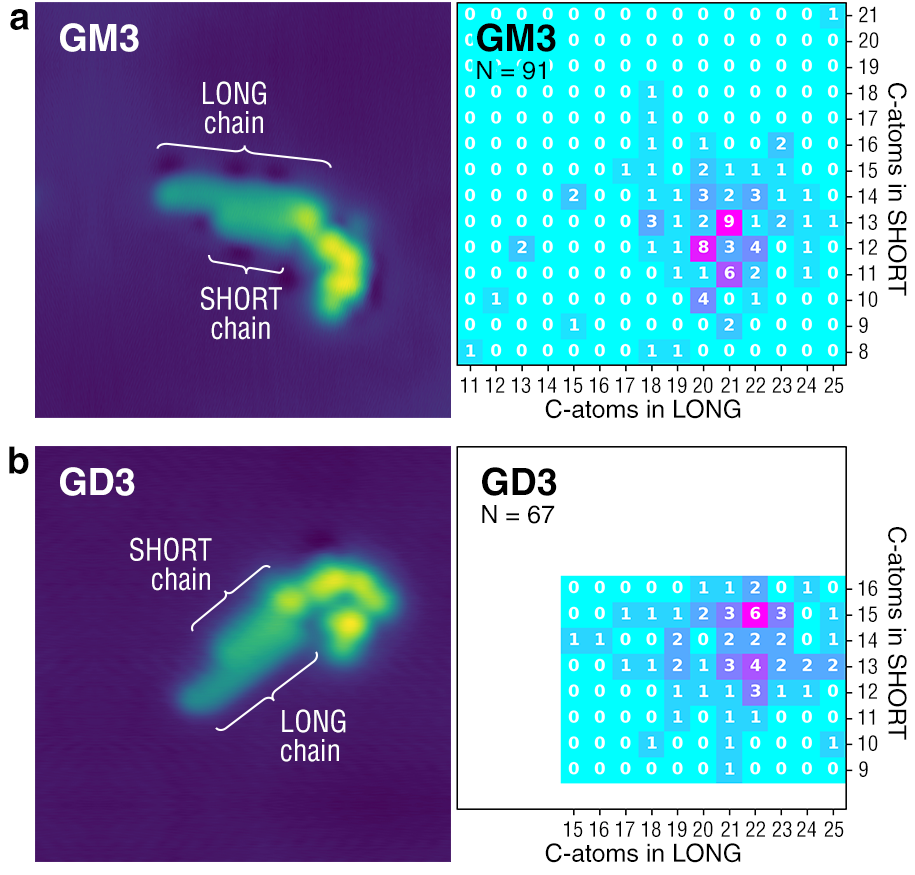


**Fig. S5 | Measured length distribution of lipid chains in GM3 and GD3 glycolipids.** All glycolipids were observed to possess a pair of short and long polyalkane chains. The lengths of these two chains were measured to reveal a correlation between them as shown by the 2D histogram plots in (**a**) for GM3 and (**b**) for GD3. The number of C-atoms in every polyalkane chain was approximated by using the value of 0.12 nm per C-atom. Each STM image is 8 × 8 nm^2^ in size.


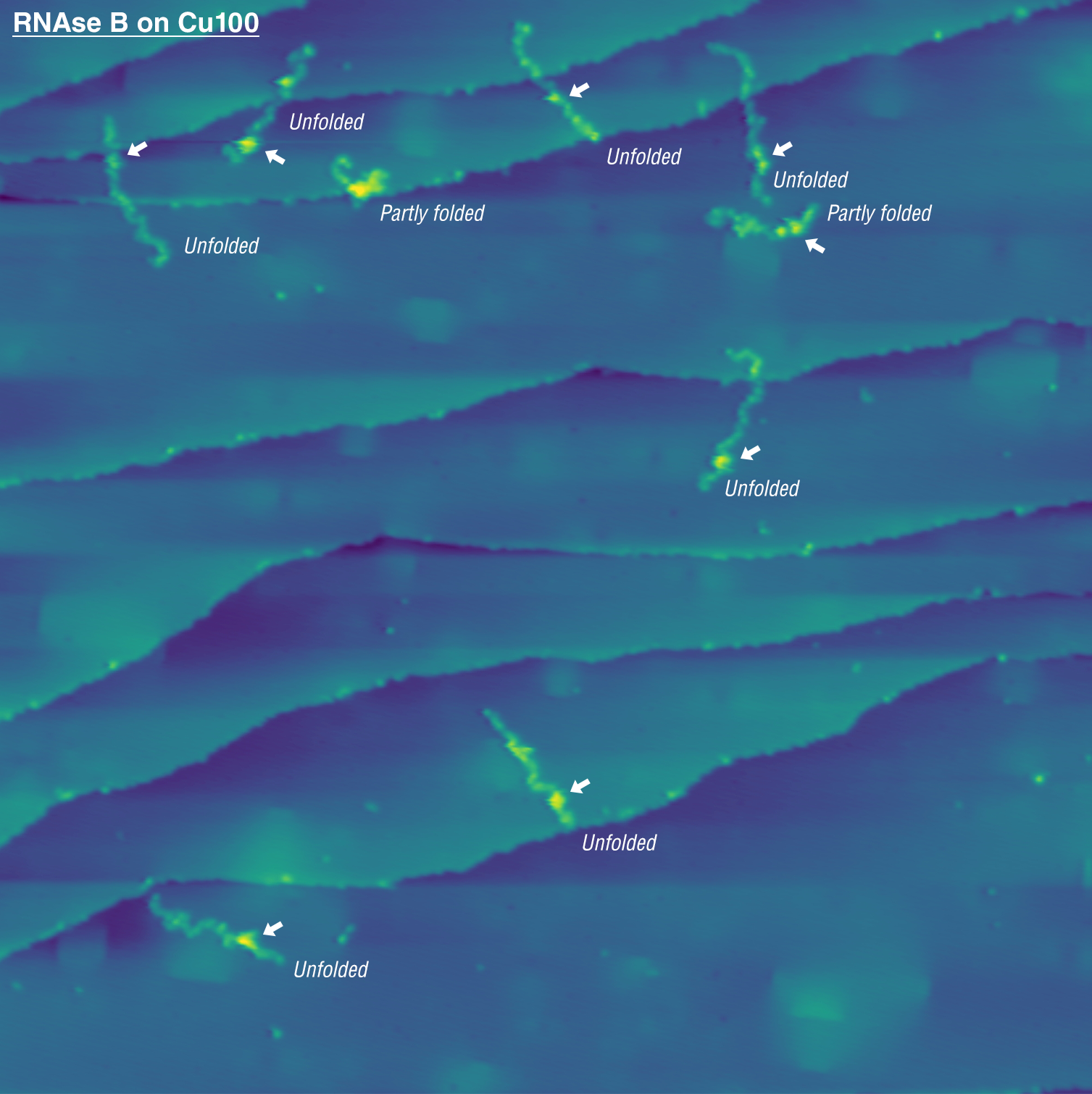


**Fig. S6 | Conformations of RNase B glycoproteins on surface.** Highly charged RNase B protein ions (+16 to +18) deposited on surface were predominantly observed in fully unfolded conformations (see Fig. S1g for the mass spectrum of the ion beam). The approximate position of the N-linked glycan on the protein is indicated by the white arrows. The landing energy employed in this specific sample was 8 – 9.5 eV (4 – 5 meV per atom). Image size is 200 × 200 nm^2^.


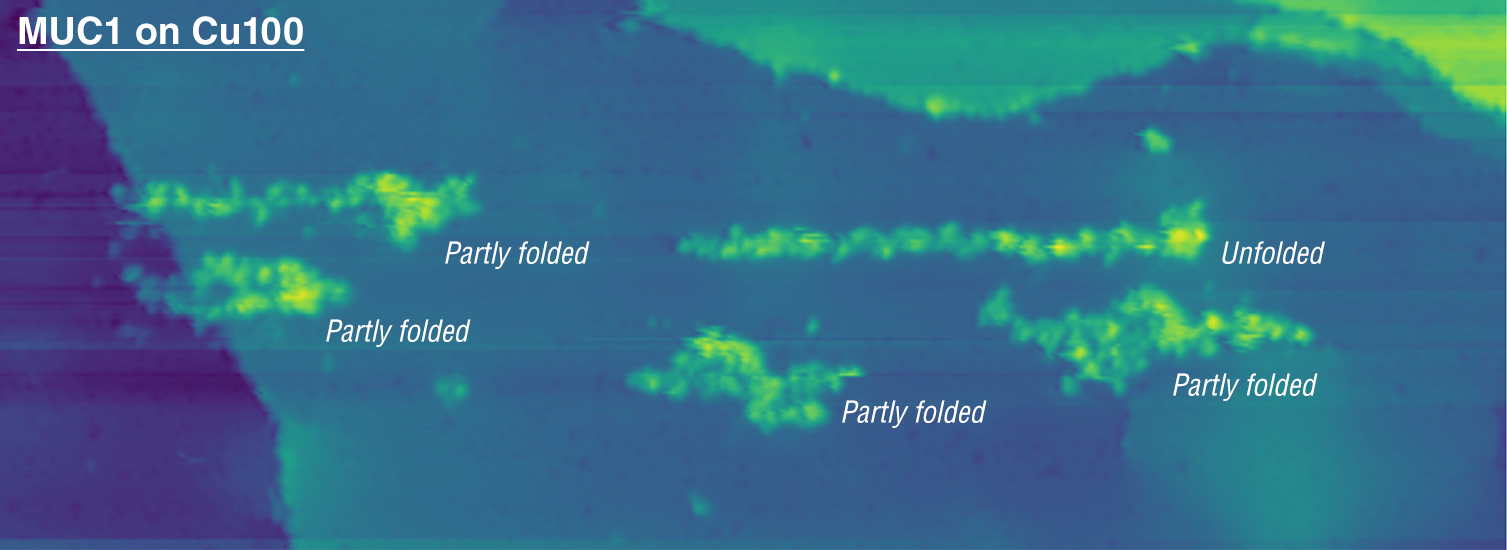


**Fig. S7 | Conformations of MUC1 glycoprotein reporters on surface.** MUC1 glycoproteins soft landed on surface were observed mainly as partly folded species. To facilitate primary structure determination of MUC1, we have mainly performed detailed imaging on the fully unfolded conformations of MUC1 on surface. Image size is 150 × 55 nm^2^.


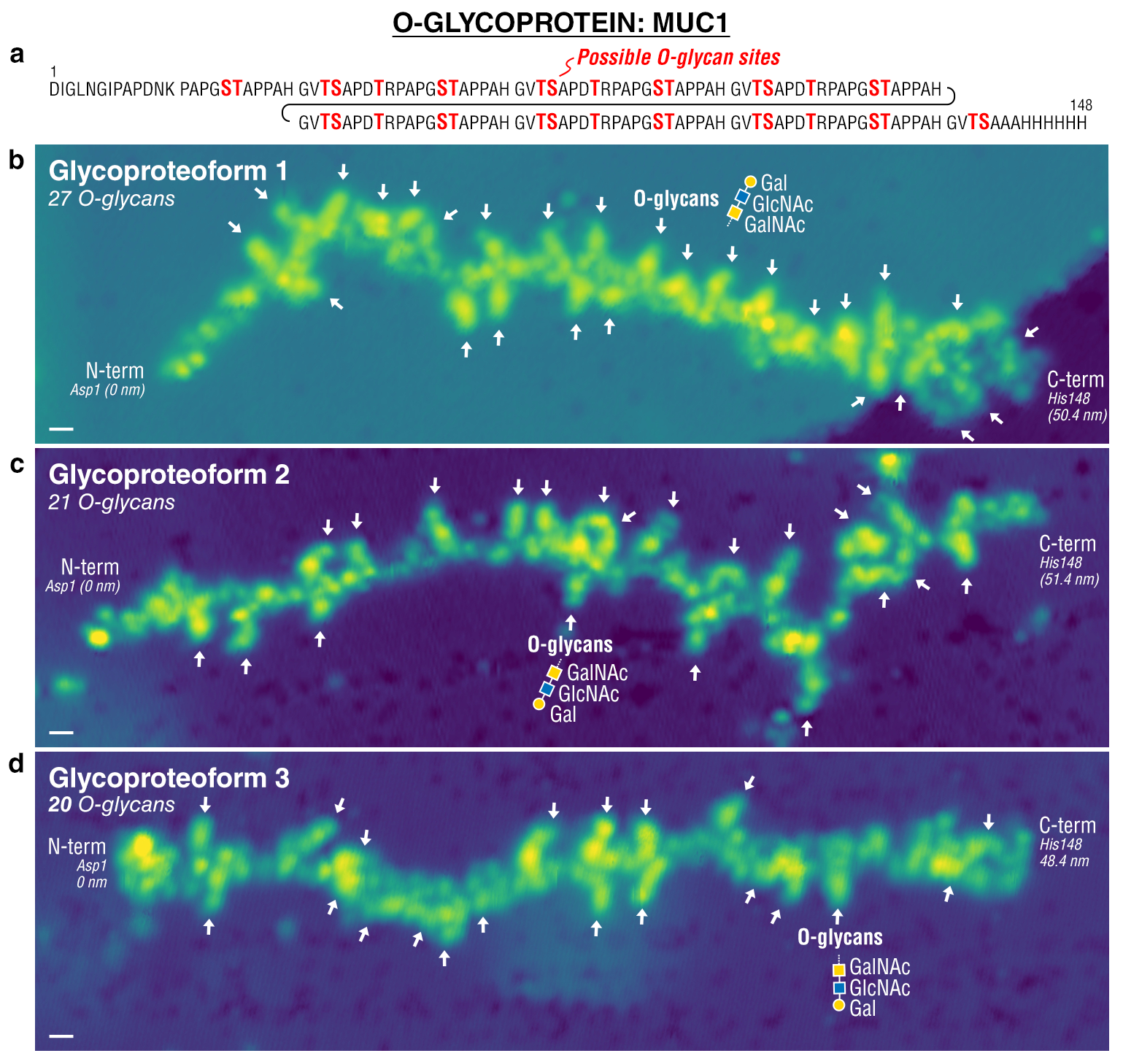


**Fig. S8 | Unannotated STM images of MUC1 glycoproteins on surface.** The arrows mark the O-glycans present along the MUC1 protein backbone. Scale bar is 1 nm.


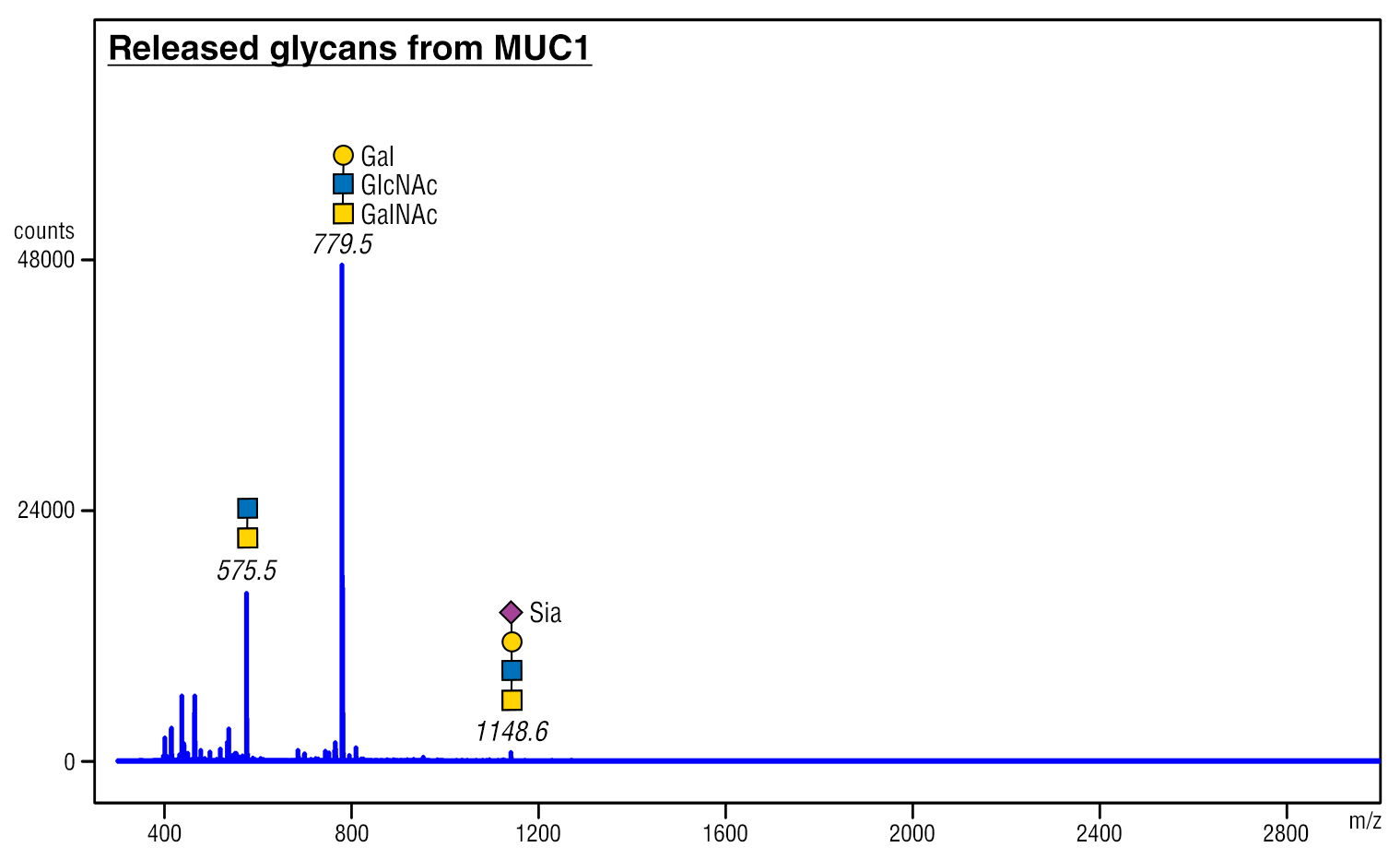


**Fig. S9 | Released O-glycoprofile from the MUC1 reporter O-glycoprotein.** MALDI mass spectrometric analysis of O-glycans released from the MUC1 O-glycoprotein expressed in HEK293 ^KO COSMC / KI B3GNT6^ cells shows predominantly core 3 trisaccharide O-glycans (Gal-GlcNAc-GalNAc), consistent with previous studies (*38*). Minor components of sialylated core 3 tetrasaccharide (Sia-Gal-GlcNAc-GalNAc) were observed in STM imaging of MUC1 (Fig. 4). Sia refers to Neu5Ac.


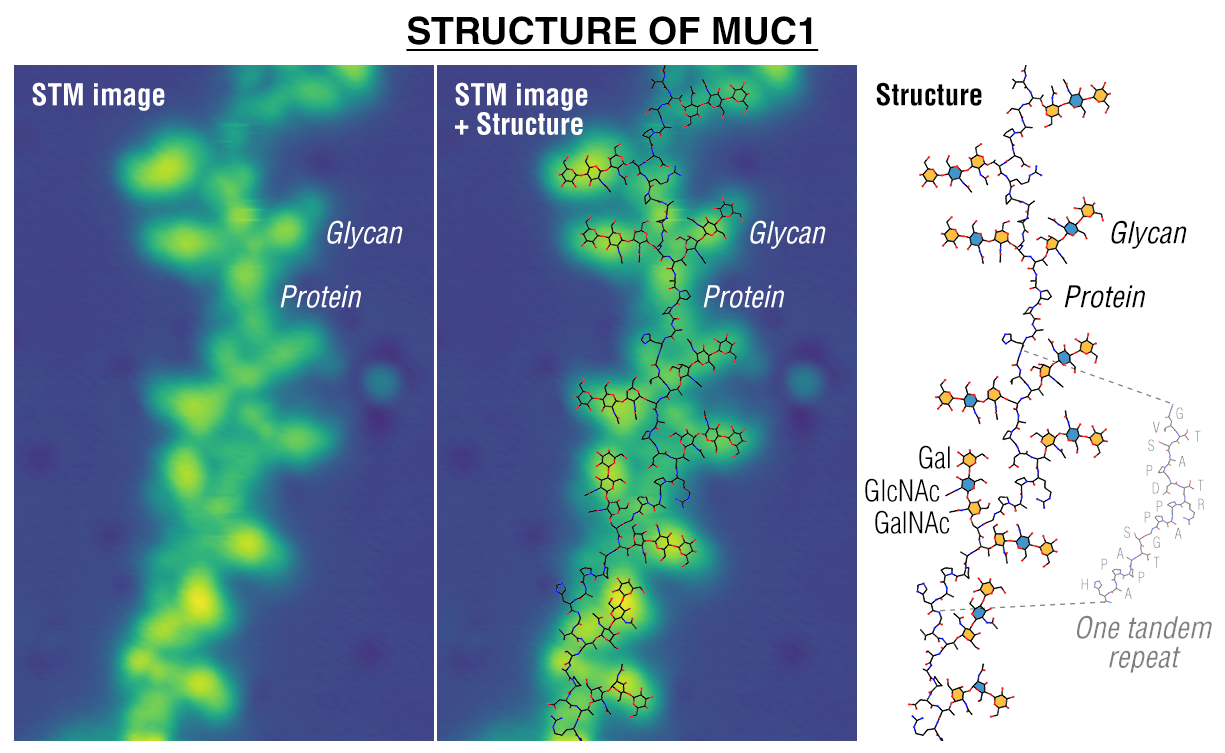


**Fig. S10 | Structure of MUC1 glycoprotein on surface.** A zoomed-in STM image of MUC1 showing the glycan and protein moieties was interpreted to yield molecular structure of MUC1 on surface.


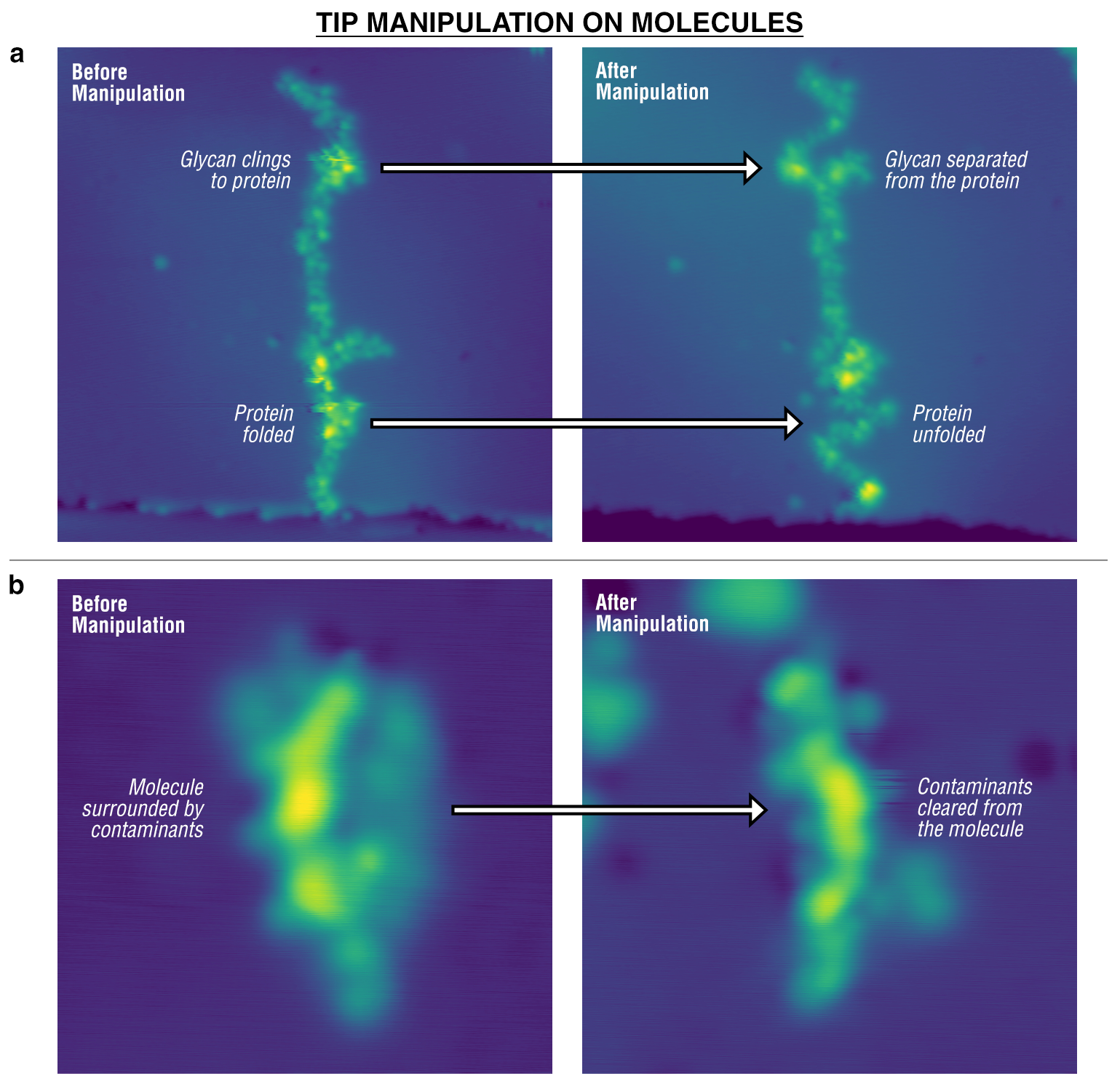


**Fig. S11 | Examples of tip manipulation on molecules.** Moving the tip in the fast-scan direction with increased setpoint (0.1 to 1 nA) may cause the molecule to intermittently latch onto the tip, causing a part of the molecule to be dragged with the tip. This operation, as exemplified in (**a**) for an RNAseB protein on Cu(100) surface, could change the conformation of the molecule, such as ‘peeling off’ parts of a glycan bound non-covalently to the protein backbone, as well as changing the protein conformation on the surface. In addition, this operation, as exemplified in (**b**) for a HS-GAG oligosaccharide (Fig S2b) on Ag(110) surface, could remove molecules (e.g. water) non-covalently bound to a molecule.

**Supplementary Tables**

| **MUC1 Sequence** | **Data** | | | | | | | | | | | | | | | | | |
| --- | --- | --- | --- | --- | --- | --- | --- | --- | --- | --- | --- | --- | --- | --- | --- | --- | --- | --- |
|  | #1 | #2 | #3 | #4 | #5 | #6 | #7 | #8 | #9 | #10 | #11 | #12 | #13 | #14 | #15 | #16 | #17 | #18 |
| DIGLNGIPAPDNKPAPG |  |  |  |  |  |  |  |  |  |  |  |  |  |  |  |  |  |  |
| **S** | O | X | O | O | O | X | O | O | X | O | X | X | O | O | O | X | O | X |
| **T** | O | O | O | O | O | A | O | O | O | O | A | O | O | O | O | O | O | A |
| APPAH |  |  |  |  |  |  |  |  |  |  |  |  |  |  |  |  |  |  |
| GV | *Tandem Repeat #1* | | | | | | | | | | | | | | | | | |
| **T** | O | X | O | X | O | O | O | O | A | A | O | O | O | O | O | O | O | A |
| **S** | O | O | X | X | X | X | O | X | A | A | X | O | X | X | X | O | X | A |
| APD |  |  |  |  |  |  |  |  |  |  |  |  |  |  |  |  |  |  |
| **T** | X | X | O | O | O | X | X | O | A | A | O | X | O | X | O | X | X | A |
| RPAPG |  |  |  |  |  |  |  |  |  |  |  |  |  |  |  |  |  |  |
| **S** | O | O | O | O | X | O | O | O | A | A | O | O | O | O | O | O | X | A |
| **T** | O | O | O | O | O | O | O | O | A | A | X | X | X | X | O | O | O | A |
| APPAH |  |  |  |  |  |  |  |  |  |  |  |  |  |  |  |  |  |  |
| GV | *Tandem Repeat #2* | | | | | | | | | | | | | | | | | |
| **T** | O | O | O | O | O | O | X | O | A | O | O | O | O | O | O | O | X | O |
| **S** | X | X | X | X | X | X | X | O | A | X | O | X | O | X | O | X | X | O |
| APD |  |  |  |  |  |  |  |  |  |  |  |  |  |  |  |  |  |  |
| **T** | O | X | O | O | X | X | X | X | X | X | O | O | O | O | X | O | O | X |
| RPAPG |  |  |  |  |  |  |  |  |  |  |  |  |  |  |  |  |  |  |
| **S** | O | O | O | O | O | O | X | O | O | O | X | O | X | O | O | X | X | O |
| **T** | O | X | O | O | O | O | O | O | O | O | X | O | O | O | O | O | O | O |
| APPAH |  |  |  |  |  |  |  |  |  |  |  |  |  |  |  |  |  |  |
| GV | *Tandem Repeat #3* | | | | | | | | | | | | | | | | | |
| **T** | O | O | O | X | O | O | O | O | O | O | O | X | O | X | A | O | O | O |
| **S** | O | O | O | X | O | O | X | X | O | X | O | X | O | X | A | X | X | O |
| APD |  |  |  |  |  |  |  |  |  |  |  |  |  |  |  |  |  |  |
| **T** | O | O | O | O | X | O | O | O | X | O | X | O | X | O | X | O | O | X |
| RPAPG |  |  |  |  |  |  |  |  |  |  |  |  |  |  |  |  |  |  |
| **S** | O | O | O | O | O | O | X | O | O | O | O | O | X | X | O | A | A | O |
| **T** | O | O | O | O | O | O | X | O | X | X | O | O | O | O | O | A | A | O |
| APPAH |  |  |  |  |  |  |  |  |  |  |  |  |  |  |  |  |  |  |
| GV | *Tandem Repeat #4* | | | | | | | | | | | | | | | | | |
| **T** | O | O | A | O | O | O | O | A | O | O | X | X | O | A | O | A | O | O |
| **S** | X | X | A | O | X | O | X | A | X | X | O | X | O | A | X | A | O | O |
| APD |  |  |  |  |  |  |  |  |  |  |  |  |  |  |  |  |  |  |
| **T** | O | X | A | X | O | O | O | A | X | X | O | X | X | A | O | A | X | X |
| RPAPG |  |  |  |  |  |  |  |  |  |  |  |  |  |  |  |  |  |  |
| **S** | O | O | A | X | O | O | X | A | O | O | A | X | A | A | O | A | O | O |
| **T** | X | O | A | O | O | O | X | A | X | O | A | X | A | A | O | A | O | O |
| APPAH |  |  |  |  |  |  |  |  |  |  |  |  |  |  |  |  |  |  |
| GV | *Tandem Repeat #5* | | | | | | | | | | | | | | | | | |
| **T** | O | O | A | O | O | O | O | O | O | O | A | X | O | X | O | A | O | X |
| **S** | X | X | A | X | O | O | X | X | X | O | A | O | O | X | X | A | X | O |
| APD |  |  |  |  |  |  |  |  |  |  |  |  |  |  |  |  |  |  |
| **T** | O | X | A | O | O | X | X | O | X | X | X | X | X | O | X | A | X | X |
| RPAPG |  |  |  |  |  |  |  |  |  |  |  |  |  |  |  |  |  |  |
| **S** | O | O | A | X | O | O | O | X | O | O | O | O | O | O | X | A | O | X |
| **T** | O | X | A | O | O | O | O | O | O | O | O | O | X | O | O | A | O | O |
| APPAH |  |  |  |  |  |  |  |  |  |  |  |  |  |  |  |  |  |  |
| GV | *Tandem Repeat #6* | | | | | | | | | | | | | | | | | |
| **T** | O | X | A | X | O | O | O | O | A | O | O | O | O | O | A | A | O | O |
| **S** | O | O | A | X | O | O | O | O | A | X | O | X | O | X | A | A | X | O |
| APD |  |  |  |  |  |  |  |  |  |  |  |  |  |  |  |  |  |  |
| **T** | O | O | O | O | O | X | X | O | A | X | X | X | O | O | A | A | O | X |
| RPAPG |  |  |  |  |  |  |  |  |  |  |  |  |  |  |  |  |  |  |
| **S** | O | O | X | X | O | O | O | X | A | O | O | O | X | X | A | A | O | O |
| **T** | X | O | O | O | O | O | O | O | A | O | O | O | O | O | A | A | X | O |
| APPAH |  |  |  |  |  |  |  |  |  |  |  |  |  |  |  |  |  |  |
| GV |  |  |  |  |  |  |  |  |  |  |  |  |  |  |  |  |  |  |
| **T** | O | O | O | X | O | O | O | A | A | O | O | O | O | O | A | A | O | O |
| **S** | X | X | O | X | O | X | O | A | A | O | X | O | O | O | A | A | O | O |
| AAAHHHHHH |  |  |  |  |  |  |  |  |  |  |  |  |  |  |  |  |  |  |

**Table S1 | Positions of O-glycans on individual MUC1 reporter O-glycoprotein molecules.** Data were obtained from observations of 18 MUC1 proteins on the surface. The letter ‘O’ marks the presence of O-glycans on their respective S or T sites; while ‘X’ marks the absence of an O-glycan. The T sites are highlighted in orange, while the S sites are highlighted in light blue. Positioning of O-glycans to their S or T sites are in most cases unambiguous, but in few sites the positioning is ambiguous (marked by the letter ‘A’ in the table) due to the O-glycan proximity to other molecular features, as well as the partial folding of the glycan or protein (see Fig. S7 for example). The tandem repeats containing ‘A’ are excluded from the population analysis presented in Table S2. Data #1, #2, and #4 are shown in Fig. 4b, 4c, and 4d respectively.

| Tandem repeat observed  (red and bold = GLYCOSYLATED) | N |
| --- | --- |
| GV**TS**APDTRPAPG**ST**APPAH | 17 |
| GV**T**SAPD**T**RPAPG**ST**APPAH | 12 |
| GV**T**SAPDTRPAPG**ST**APPAH | 11 |
| GV**TS**APD**T**RPAPG**ST**APPAH | 7 |
| GV**T**SAPD**T**RPAPGS**T**APPAH | 5 |
| GV**T**SAPD**T**RPAPG**S**TAPPAH | 5 |
| GVTSAPD**T**RPAPG**ST**APPAH | 4 |
| GV**T**SAPDTRPAPG**S**TAPPAH | 4 |
| GV**TS**APDTRPAPG**S**TAPPAH | 3 |
| GV**TS**APD**T**RPAPGS**T**APPAH | 3 |

**Table S2 | Top 10 most common O-glycosylation pattern in a tandem repeat observed in MUC1.** Data was obtained from Table S1. The top three most common structures reported in the main text are highlighted in light blue. Interestingly, single O-glycans in these TS/ST motifs were found not only on the T residues as found by bottom-up analysis (*38*), but also in some cases on the S residues (e.g. in the TS sequon of first repeat sequence in the glycoproteoform with 21 O-glycans shown in Fig. 4b). In addition, the data also suggests a correlation between different glycan sites which may inform how the protein is decorated with glycans. For example, on the triply glycosylated GV**T***S*APD*T*RPAPG**ST**APPAH, the fourth O-glycan is ~1.4 times more likely to be assembled at the *S* of V**T***S*A than the *T* of PD*T*R – because GV**TS**APDTRPAPG**ST**APPAH (*N* = 17) was ~1.4 times more likely to be observed than GV**T**SAPD**T**RPAPG**ST**APPAH (*N* = 12). Similarly, on the doubly glycosylated GV**T**SAPD*T*RPAPG*S***T**APPAH, the third O-glycan is ~2.2 times more likely to be assembled at the *S* of G*S***T**A than the *T* of PD*T*R.
